# Applicability of the Environmental Relative Moldiness Index for Quantification of Residential Mold Contamination in an Air Pollution Health Effects Study

**DOI:** 10.1155/2014/261357

**Published:** 2014-11-09

**Authors:** Ali Kamal, Janet Burke, Stephen Vesper, Stuart Batterman, Alan Vette, Christopher Godwin, Marina Chavez-Camarena, Gary Norris

**Affiliations:** ^1^National Exposure Research Laboratory, United States Environmental Protection Agency, 109 T.W. Alexander Drive, Research Triangle Park, NC 27711, USA; ^2^National Exposure Research Laboratory, United States Environmental Protection Agency, 26 West M. L. King Drive, Cincinnati, OH 45268, USA; ^3^Department of Environmental Health Sciences, School of Public Health, University of Michigan, 1420 Washington Heights, Ann Arbor, MI 48109, USA; ^4^Community Action Against Asthma Partner, Detroit, MI 48202, USA

## Abstract

The Near-Road Exposures and Effects of Urban Air Pollutants Study (NEXUS) investigated the impact of exposure to traffic-related air pollution on the respiratory health of asthmatic children in Detroit, Michigan. Since indoor mold exposure may also contribute to asthma, floor dust samples were collected in participants homes (*n* = 112) to assess mold contamination using the Environmental Relative Moldiness Index (ERMI). The repeatability of the ERMI over time, as well as ERMI differences between rooms and dust collection methods, was evaluated for insights into the application of the ERMI metric. ERMI values for the standard settled floor dust samples had a mean ± standard deviation of 14.5 ± 7.9, indicating high levels of mold contamination. ERMI values for samples collected from the same home 1 to 7 months apart (*n* = 52) were consistent and without systematic bias. ERMI values for separate bedroom and living room samples were highly correlated (*r* = 0.69, *n* = 66). Vacuum bag dust ERMI values were lower than for floor dust but correlated (*r* = 0.58, *n* = 28). These results support the use of the ERMI to evaluate residential mold exposure as a confounder in air pollution health effects studies.

## 1. Introduction

Asthma is the most common chronic disease of children in the United States (US) [1, 2]. Asthma prevalence and related death rates in Detroit are the highest in Michigan and among the highest in the US [3]. Living close to busy roads may be an important risk factor for onset of childhood asthma, and studies have found positive associations between exposure to traffic-related pollution and wheezing in children [4]. In addition, exposure of children in Detroit to ambient air pollutants has been associated with asthma exacerbation [5–7]. Currently, the Near-Road Exposures and Effects of Urban Air Pollutants Study (NEXUS) is investigating the impact of exposure to traffic-related air pollutants on the respiratory health of a cohort of children with asthma who live near major roadways in Detroit [8]. However, other exposures may also contribute to asthma-related health outcomes, including cigarette smoke, allergens, and mold [9–13]. An assessment of residential mold contamination was, therefore, included in the NEXUS study design.

To standardize the quantification of mold contamination in homes, the Environmental Relative Moldiness Index (ERMI) scale was createdby US EPA researchers in conjunction with the US Department of Housing and Urban Development (HUD) [14]. The standard sample for an ERMI analysis is a composite of bedroom and living room floor dust obtained using the MiTest sampler. This sample is analyzed using a DNA-based method, mold specific quantitative polymerase chain reaction (MSQPCR), to quantify the concentration of 36 indicator molds used to determine the ERMI [14]. The ERMI provides a metric for the relative amount of mold due to water damage compared to other ubiquitous molds. The ERMI scale has proven to be a useful metric for understanding the role of mold exposure in asthma development [15, 16].

In this study, we sought to understand the applicability of the ERMI for quantification of residential mold contamination by evaluating the stability of the ERMI for a home over time; differences in the ERMI for living room versus bedroom samples compared to the standard composite sample; and whether vacuum cleaner bag dust could provide meaningful information relative to the standard ERMI dust sample. Lastly, we compared ERMI values and traffic exposure groups for NEXUS homes to understand whether exposure to mold in the home may be an important confounder in this study.

## 2. Methods

### 2.1. Study Recruitment and Design

The NEXUS study population consisted of children aged 6–14 years with symptoms consistent with asthma and living in Detroit, Michigan, that were identified using community-based screening methods, including door-to-door canvassing [8]. The selection of families was based on distance of their home from major highways and report of spending multiple nights per week at their primary residence. After a preliminary determination of eligibility by questionnaire, a subset of those living in each of the exposure zones of interest were selected to participate in a “wash in” evaluation to teach and assess adherence to study protocols including spirometry technique. The enrollment rate was 77% of those who completed the wash-in process. There was no difference in rate of enrollment among people living in the three highway exposure zones.

Recruitment and study protocols utilized written informed consent and followed ethical guidelines approved by the University of Michigan Institutional Review Board and our community-based partners. NEXUS participants were recruited based on the proximity of their home to major roadways with different traffic characteristics. Each home was assigned to one of the following traffic categories: high traffic/high diesel (HT/HD), high traffic/low diesel (HT/LD), and low traffic/low diesel (LT/LD) [8].

### 2.2. Dust Sampling and Mold Analysis

Settled floor dust samples were collected from 112 NEXUS participant homes for mold analysis from October 2010 through April 2012. Settled floor dust samples were collected by vacuuming a 2 m^2^ area for five minutes with a MiTest sampler (Indoor Biotechnologies, Charlottesville, VA) in the rooms, as described below. The MiTest sampler contains a filter with a 40 *μ*m pore size but in use the effective pore size is reduced by the accumulation of dust so that even very small particles like allergens are captured. For 66 of 112 homes, a floor dust sample was obtained from the bedroom and a separate one also from the living room. These samples were kept separate for analysis. The remaining bedroom and living room dust from these samples were composited to create the “standard” ERMI dust sample which is a single sample from both the bedroom and living room. For the other 46 of the 112 homes, only the standard ERMI sample (bedroom plus living room) was obtained [14].

For 52 participants' homes, the standard dust sample collection was repeated, one to seven months apart, to characterize potential variability over the duration of the study. Also, dust samples from household vacuum cleaner bags were collected from a total of 33 homes, 28 of which had a standard settled floor dust sample for comparison.

The dust samples were kept at room temperature in the dark until about 12 were accumulated and then these were shipped overnight to the laboratory for analysis. Each dust sample was then frozen at −20°C until it was sieved through a 300 *μ*m pore size nylon mesh (Gilson Company, Inc. Lewis Center, OH) and then 5.0 ± 0.1 mg of sieved dust was extracted and the DNA purified using the DNA-EZ extraction kit (GeneRite, Cherry Hill, NJ), following the manufacturer's instructions. These extracts were frozen at −20°C until analyzed. Methods and assays have previously been reported for performing MSQPCR analyses [17] to obtain mold concentrations for calculation of the ERMI [14].

### 2.3. Determination of ERMI Values

The ERMI is calculated as the difference between the log concentrations of 26 mold species associated with water damage (Group 1) and the log concentrations of 10 species commonly found in homes without water damage (Group 2):

(1) $$\begin{array}{c} \text{ERMI}=\overset{26}{\underset{i=1}{\sum }}\text{lo}{\text{g}}_{10}\left(s_{1i}\right)-\overset{10}{\underset{j=1}{\sum }}\text{lo}{\text{g}}_{10}\left(s_{2j}\right), \end{array}$$

where *s*_1*i*_ and *s*_2*j*_ are concentrations of Groups 1 and 2 molds, respectively [14]. The ERMI typically ranges from −10 to 20; however, ERMI values greater than 20 have been found in highly contaminated homes. An ERMI value greater than 5 is in the upper quartile (highest mold contamination quartile) for homes in the US [14].

### 2.4. Statistical Analyses

The Bland and Altman plot was created in Sigma Plot (Systat Software, Inc. San Jose, CA). All other statistical summaries and comparisons were performed with SAS software (Cary, NC) including linear regression, Spearman correlation, and Kolmogorov-Smirnov (KS) tests.

## 3. Results

### 3.1. ERMI for NEXUS Homes

The ERMI values for the standard dust samples collected from NEXUS participant homes (*n* = 112) had a mean ± standard deviation (SD) of 14.5 ± 7.9, and ranged from −2.5 to 33.9 (Table 1). Most of the homes (85%) had an ERMI value greater than 5, the upper quartile of ERMI values for homes in the US, and 26% had an ERMI value over 20, indicating a high level of residential mold contamination in the NEXUS homes. Summary statistics for concentrations of the 36 mold species used for the ERMI are provided for the floor dust samples (Table 2).

### 3.2. ERMI Values Compared over Time

**Figure 1:**
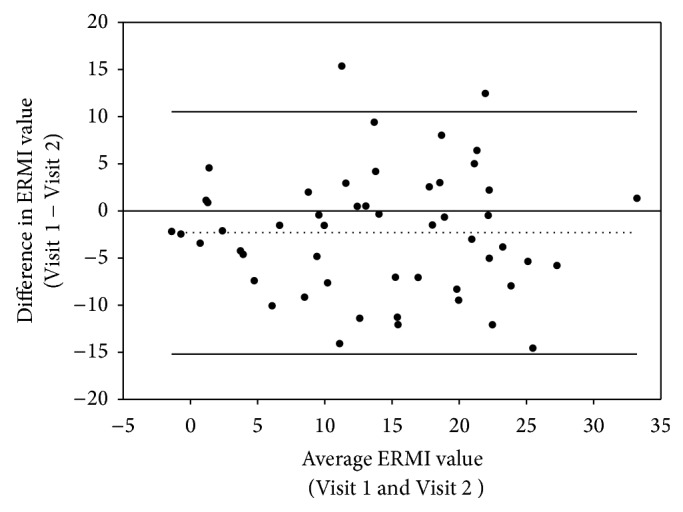
Comparison of Environmental Relative Moldiness Index (ERMI) values for the initial (Visit 1) and repeat (Visit 2) floor dust samples from NEXUS homes (*n* = 52) in a Bland and Altman plot of difference versus average. Dotted line is the mean difference and solid lines indicate the 95% confidence interval.

The mean ± SD of ERMI values for the initial and repeat settled floor dust samples collected from the same home 1 to 7 months apart were 12.8 ± 8.8 and 15.2 ± 9.0, respectively (Table 1). The repeatability of the ERMI measurements was assessed using a Bland and Altman plot (Figure 1). The average ERMI value (on *x*-axis) is plotted against the difference (on *y*-axis) for all 52 homes with repeated sampling. The average difference was −2.3, and 50 of the 52 measurements were within the 95% confidence interval indicating a strong likelihood of repeatability of ERMI composite measurements without any systematic bias in the results.

The largest differences in ERMI values between initial and repeat samples were primarily due to differences in Group 1 molds (MAD = 7.5 ± 6.1), as Group 2 molds had smaller differences between samples (MAD = 3.1 ± 2.7) (Table 1). However, Group 1 molds were more highly correlated between the initial and repeat samples than Group 2 molds, but correlation was highest for the ERMI values (Table 1).

To identify possible determinants of the variability in Figure 1, differences in ERMI values between repeat samples for the same home were compared by the month and season each sample was collected, as well as by the number of days between samples. No relationship was found that explained a significant proportion of the observed differences in ERMI values. However, when divided into three groups based on the number of days between initial and repeat samples (22 to 210, median = 112 days), the correlation increased and MAD decreased when the repeat sample was collected within 90 days of the initial sample (Spearman *r* = 0.76, MAD = 4.9, *n* = 15). Correlation was lower and MAD higher for repeat samples collected 90–180 days after the initial sample (Spearman *r* = 0.67, MAD = 5.4, *n* = 28) or 180–210 days after the initial sample (Spearman *r* = 0.62, MAD = 6.7, *n* = 9).

### 3.3. ERMI Values for Bedroom versus Living Room

**Figure 2:**
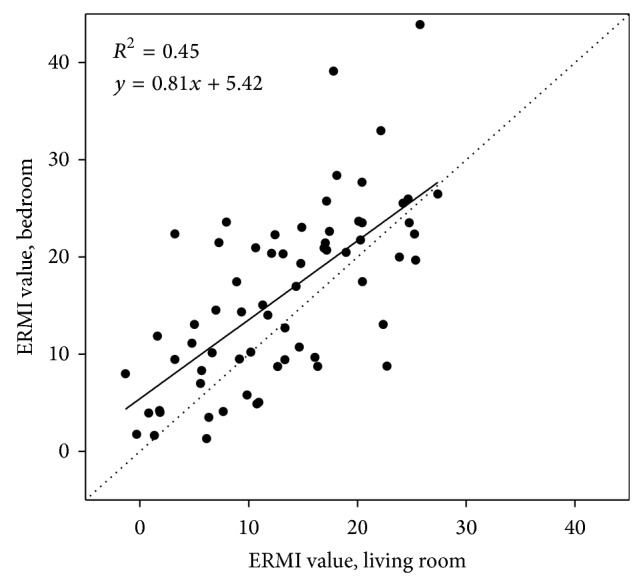
Comparison of Environmental Relative Moldiness Index (ERMI) values for separate bedroom and living room settled floor dust samples from NEXUS homes (*n* = 66). The black solid line is a linear fit of all the data.

For homes with separate bedroom and living room floor dust samples, the mean ERMI for bedrooms (16.1 ± 9.1) was typically higher (but not significantly) than for living rooms (13.1 ± 7.6) with a MAD of 5.8. However, ERMI values were significantly correlated (Spearman *r* = 0.69; *P* < 0.001) between rooms within the home.  Figure 2 indicates a linear relationship between living room and bedroom ERMI values although with a high degree of variability. Approximately 20% of the homes had similar ERMI values for both rooms (differed by <2), while ERMI values differed by 10 or more between rooms for another 20% of homes. Both Group 1 and Group 2 molds had similar patterns and correlation between rooms as for the ERMI values (Table 1).

### 3.4. ERMI Values for Composite Dust Samples versus Vacuum Bag Dust

**Figure 3:**
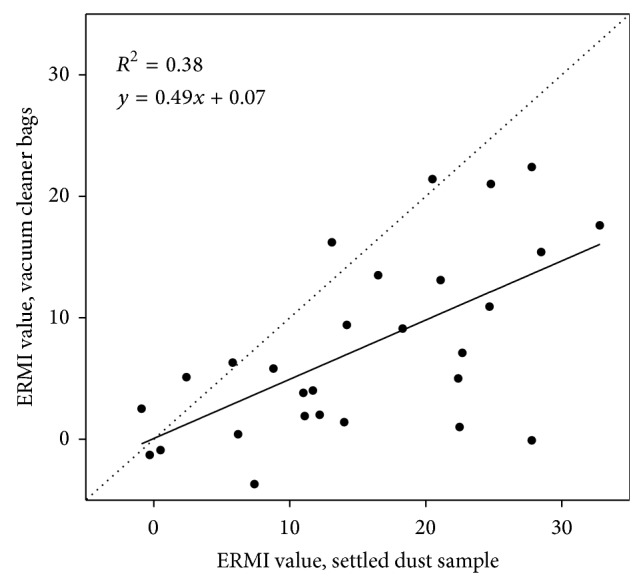
Comparison of Environmental Relative Moldiness Index (ERMI) values for settled floor dust samples and household vacuum bag dust from NEXUS homes (*n* = 28). The black solid line is a linear fit of the data.

ERMI values for the standard composite settled floor dust samples and vacuum bag dust samples from the same home were moderately correlated (Spearman *r* = 0.58; *P* = 0.001) with a linear relationship (Figure 3). However, ERMI values from composite dust samples were nearly twice as high on average as the vacuum bag dust ERMI values, 15.3 ± 9.5 versus 7.5 ± 7.5 (Table 1). Group 1 molds were more strongly correlated (Spearman *r* = 0.70; *P* < 0.001) for the two types of samples than for Group 2 ubiquitous mold species (Spearman *r* = 0.42; *P* = 0.03).

### 3.5. ERMI Values in Homes for Different Traffic Classification

**Figure 4:**
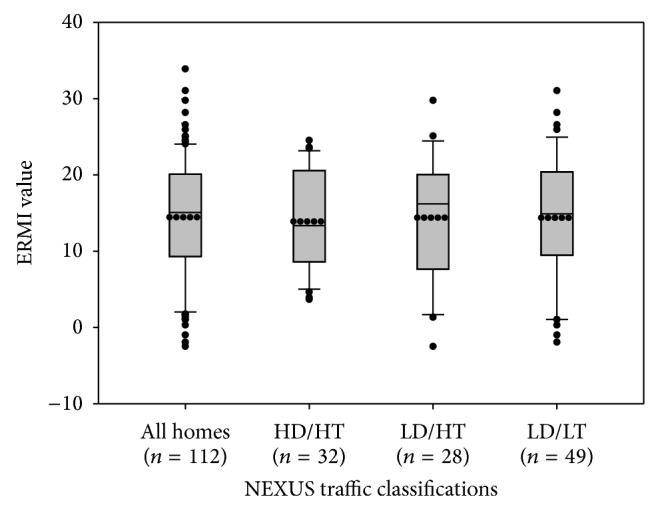
Distribution of Environmental Relative Moldiness Index (ERMI) values of settled floor dust by traffic classification: high traffic/high diesel (HT/HD), high traffic/low diesel (HT/LD), and low traffic/low diesel (LT/LD). The mean is represented by the dotted line. (Three of the homes were classified with moderate diesel exposures and not included in the classification analysis.)

The distributions of ERMI values for the standard settled floor dust samples were similar among the three main traffic classifications for the NEXUS homes (Figure 4). Mean ERMI values were 13.9 ± 6.5, 14.4 ± 8.3, and 14.4 ± 8.3 for homes in the HD/HT, LD/HT, and LD/LT groups, respectively, and their distributions were not statistically different (Kolmogorov-Smirnov test: KS = 0.79). This suggests that the ERMI values are independent of the NEXUS traffic exposure classifications which were based on each home's proximity to major highways.

## 4. Discussion

The asthmatic children in Detroit that participated in NEXUS had homes with high levels of mold contamination compared to previous studies that also used the standard ERMI settled floor dust samples. The mean ERMI for NEXUS homes was 14.5 compared to 6.7 for homes of asthmatic children in Cincinnati, OH [16] and 8.7 for homes of asthmatic children in three US cities: Kansas City, KS, Boston, MA, and San Diego, CA [18]. These studies also found that homes of asthmatic children had ERMI values twice as high on average as homes of children without asthma or randomly sampled control homes. Clearly, the majority of NEXUS participants' homes were highly contaminated by mold. High levels of mold contamination have been associated with older, urban housing stock in other cities [14, 18].

Many methods and techniques have been used to quantify mold contamination but the most common is a very short air sample from which the molds are quantified by counting spores under a microscope or culturing on specific media. These short air samples are now widely recognized for their limitations [19, 20]. In this study, we examined whether the standard composite ERMI dust sample provided reasonably consistent estimates of mold contamination in a home over a period of months based on repeat samples from the same home. Although ERMI values were generally consistent with no clear bias, conclusions were limited by the large differences between samples for many NEXUS homes. However, the stronger correlation and smaller differences between samples collected less than 3 months apart provide additional support for applicability of the ERMI over the study period. It is possible that the high levels of mold contamination in the NEXUS homes contributed to the observed variability in the ERMI over time. Future analyses with the health effects data may help determine whether large differences over time for homes that are high on the ERMI scale (i.e., 10 versus 25) are meaningful in the context of this study.

The results indicate that the ERMI for the standard composite settled floor dust sample is an appropriate metric for overall mold contamination in the NEXUS homes when compared to separate bedroom and living room floor dust samples or household vacuum bag dust. While ERMI values for the separate bedroom and living room samples differed substantially for many homes, they were significantly correlated and neither room type had consistently higher or lower ERMI values across the homes. In addition, the homeowner's vacuum bag dust usually provided a much lower ERMI value than the standard composite sample of living room and bedroom settled floor dust. However, these lower ERMI values for vacuum bag dust samples in NEXUS (mean = 7.6) were similar in magnitude to a previous study that compared ERMI values for vacuum bag dust from homes of children with severe asthma (mean = 8.2) to homes of children without asthma (mean = 6.2) in Detroit [21].

Although it would be desirable to be able to monitor all exposures continuously during an epidemiological study like NEXUS, for many exposures, including mold, this is not practical. However, other epidemiological studies have successfully utilized the ERMI metric to estimate mold exposures and have been able to demonstrate predictive relationships between mold exposure estimates and asthma. For example, in a prospective study of the development of asthma, infants' exposure to high ERMI homes was the only exposure predictive of the age seven diagnosis of asthma [15]. The relative risk of an infant developing asthma nearly doubled for each 10-unit increase in the home's ERMI value [16]. In Kansas City, severely asthmatic children lived in homes with significantly higher ERMI values than those with mild to moderate asthma [18]. So the ERMI metric, calculated from a composite of the living room and bedroom dust, has been found useful in studies of childhood asthma.

On the other hand, ERMI values based on household vacuum bag samples were considerably lower than those from the composite floor dust samples from the same home. This might be expected since there is no standardization or control over what ends up in a homeowner's vacuum bag. In addition, the mold cells are likely diluted with other particles captured by the vacuum cleaner. However, we found moderate correlation between ERMI values from household vacuum bag and composite dust samples. Similar trends were seen in a study of the microbial content of house dust for 5 homes in Finland [22]. A previous study using vacuum bag dust found that severely asthmatic children lived in homes with significantly higher ERMI values than those with mild to moderate asthma [21]. Therefore, mold measurements from household vacuum bag dust samples may provide useful information for comparing mold contamination between homes, but these ERMI values will not be the same as the standard composite dust sample's ERMI value.

Although the NEXUS study is focused on traffic sources of air pollution and their impact on the respiratory health of asthmatic children, there are other outdoor and indoor exposures that could confound this assessment. Other studies have shown the importance of assessing multiple exposures for asthmatic children to better understand health effects [23]. In addition, practices in the home like the use of windows for ventilation or cleaning frequency could also affect these results. The similar distributions of ERMI values for homes in the high diesel/high traffic, low diesel/high traffic, or low diesel/low traffic groups indicate that the relative mold contamination was not different in any group of homes and that the ERMI data can be used to apportion the impact of residential mold exposure on health outcomes in the NEXUS study.

However, we recognize that there are many limitations to our study of mold contamination in the NEXUS homes. For example, children do not spend all of their time in the bedroom and living room or even in their own home. School is another important source of potential exposure to mold that was not measured in the study. Also, the population size was below what was anticipated, as some families did not consent to have their homes resampled and many refused to provide a vacuum cleaner bag at the time of the floor dust sample collection. Also, the variable length of time between the initial and repeat sampling events was not ideal. A more consistent time interval or seasonal pattern between samples may have provided additional insights, but scheduling to reenter a home was often difficult and some children moved during the study period. In spite of these limitations, the results have improved our understanding of the applicability of the ERMI metric for assessing residential mold exposure in this air pollution exposure and health study of asthmatic children in Detroit.

## Figures and Tables

**Table 1:** Summary statistics of Environmental Relative Moldiness Index (ERMI) values and Groups 1 and 2 mold concentrations for dust samples collected from homes of NEXUS participants with Spearman correlation coefficient (**ρ**) for paired comparisons (^*^ *P* < 0.01).

Sample type	*N*	ERMI		Group 1 mold concentrations (log⁡CE/mg)		Group 2 mold concentrations (log⁡CE/mg)	
		Mean ± std. dev.	**ρ**	Mean ± std. dev.	**ρ**	Mean ± std. dev.	**ρ**
Settled floor dust	112	14.5 ± 7.9		35.1 ± 9.9		20.6 ± 3.7	

Initial settled floor dust	52	12.8 ± 8.8	0.71^*^	32.8 ± 11.0	0.63^*^	20.0 ± 3.8	0.36^*^
Repeat settled floor dust		15.2 ± 9.0		35.9 ± 11.5		20.7 ± 3.8	

Living room settled floor dust	66	13.1 ± 7.6	0.69^*^	32.8 ± 9.8	0.68^*^	19.7 ± 4.2	0.63^*^
Bedroom settled floor dust		16.1 ± 9.1		36.1 ± 13.0		20.1 ± 5.5	

Settled floor dust	28	15.3 ± 9.5	0.58^*^	35.8 ± 12.0	0.70^*^	20.6 ± 4.1	0.42
Household vacuum bag dust		7.5 ± 7.5		22.2 ± 10.0		14.7 ± 4.1	

**Table 2:** Mean and standard deviation (SD) of concentrations by mold species in settled floor dust samples for NEXUS homes (*n* = 112). Concentration expressed as log cell equivalents (CE) per mg.

	Mold species	Concentration (log⁡CE/mg)	
		Mean	SD
	Group 1		
1	* Aspergillus flavus *	0.57	0.52
2	* Aspergillus fumigatus *	0.88	0.49
3	* Aspergillus niger *	2.32	0.71
4	* Aspergillus ochraceus *	1.87	0.80
5	* Aspergillus penicillioides *	1.89	0.56
6	* Aspergillus restrictus *	1.78	0.41
7	* Aspergillus sclerotiorum *	1.02	0.65
8	* Aspergillus sydowii *	1.41	0.70
9	* Aspergillus unguis *	1.00	0.75
10	* Aspergillus versicolor *	1.68	0.70
11	* Aureobasidium pullulans *	4.08	0.61
12	* Chaetomium globosum *	1.21	0.71
13	* Cladosporium sphaerospermum *	1.65	0.53
14	* Eurotium *group	2.20	0.67
15	* Paecilomyces variotii *	0.95	0.69
16	* Penicillium brevicompactum *	1.67	0.61
17	* Penicillium corylophilum *	0.91	0.63
18	* Penicillium crustosum *group	1.63	0.70
19	* Penicillium purpurogenum *	0.79	0.69
20	* Penicillium spinulosum *	0.33	0.37
21	* Penicillium variabile *	0.75	0.57
22	* Scopulariopsis brevicaulis *	0.90	0.77
23	* Scopulariopsis chartarum *	1.03	0.70
24	* Stachybotrys chartarum *	1.25	0.62
25	* Trichoderma viride *	1.15	0.64
26	* Wallemia sebi *	3.41	0.67

	Group 2		
27	* Acremonium strictum *	0.68	0.42
28	* Alternaria alternata *	2.70	0.50
29	* Aspergillus ustus *	1.13	0.59
30	* Cladosporium cladosporioides* Type 1	3.61	0.42
31	* Cladosporium cladosporioides* Type 2	1.74	0.44
32	* Cladosporium herbarum *	2.82	0.39
33	* Epicoccum nigrum *	2.96	0.52
34	* Mucor *group	2.30	0.86
35	* Penicillium chrysogenum* Type 2	2.13	0.70
36	* Rhizopus stolonifer *	0.93	0.78
